# Effects of Inline Skating Exercise on Symptoms, Executive Functions, and Motor Proficiency in Children With ADHD: A Randomized Controlled Trial

**DOI:** 10.1155/oti/4254970

**Published:** 2025-05-08

**Authors:** Chu-Yang Huang, Wen-Fan Chen, Chia-Liang Tsai, Po-Lin Chen, Po-Jen Hsu, Chien-Yu Pan

**Affiliations:** ^1^Institute of Medical Science and Technology, National Sun Yat-sen University, Kaohsiung City, Taiwan; ^2^Department of Physical Education, National Kaohsiung Normal University, Kaohsiung City, Taiwan; ^3^Institute of Physical Education, Health and Leisure Studies, National Cheng Kung University, Tainan City, Taiwan; ^4^Department of Kinesiology, Mississippi State University, Mississippi State, Mississippi, USA

**Keywords:** ADHD, executive functions, inline skating, motor proficiency, symptoms

## Abstract

**Background and Purpose:** A growing body of evidence demonstrates that physical exercise training is beneficial in the treatment of attention-deficit/hyperactivity disorder (ADHD). This study is aimed at examining the effects of a 12-week inline skating intervention on ADHD symptoms, executive functions (EFs), and motor proficiency in children with ADHD.

**Methods:** This study employed an asymmetric crossover randomized controlled trial (RCT) design. A total of 24 children with ADHD (aged 6–12 years) were recruited from nongovernmental organizations, elementary schools, and parent networks. Participants were paired based on age, medication status, and ADHD presentation and then randomly assigned to either an intervention group (IG) (*n* = 12) or a wait-list control group (CG) (*n* = 12). The IG participated in a 12-week inline skating program (80-min sessions, twice weekly), while the CG maintained their regular daily routines and did not participate in any structured physical activity (PA) or skating training during this period. After 12 weeks, the CG underwent the same intervention. ADHD symptoms (parent reported), EFs (inhibition and spatial working memory), and motor proficiency (fine motor control, body coordination, manual coordination, strength, and agility) were assessed at three time points: baseline (T1), posttest (T2), and follow-up (T3). Data were analyzed using mixed-design ANOVA to evaluate the effects of the intervention.

**Results:** Children with ADHD in the current study demonstrated improved symptoms, EFs, and motor proficiency (all *p* < 0.05) after 12-week inline skating intervention. Moreover, the effects appeared to be sustained for at least 12 weeks.

**Conclusion:** This RCT provides preliminary evidence that inline skating may be a feasible and beneficial PA intervention for children with ADHD. However, larger studies are needed to further evaluate its long-term efficacy.

**Trial Registration:** Australian New Zealand Registry of Clinical Trials: ACTRN12624000593538

## 1. Introduction

Attention-deficit/hyperactivity disorder (ADHD) is a neurodevelopmental disorder that commonly emerges during early childhood. According to the *Diagnostic and Statistical Manual of Mental Disorders, Fifth Edition, Text Revision*, the core characteristics of ADHD are persistent inattention, hyperactivity, and impulsivity [1]. Depending on the diagnostic criteria, ADHD is divided into three subtypes: a predominantly inattentive subtype, a predominantly hyperactive or impulsive subtype, and a combined subtype [1]. Generally, the majority of children with ADHD are likely to continue exhibiting symptoms into adolescence [2] and adulthood [3]. Therefore, ADHD may affect the future development of these children.

In addition to the core symptoms of ADHD, individuals with the disorder often experience challenges in executive functions (EFs). EFs are thought to be comprised of three core processes (i.e., inhibition, working memory, and cognitive flexibility) [4]. Earlier research in children with ADHD described impairment in EFs, including inhibitory control, working memory, and set shifting [5]. Emerging evidence confirms that executive dysfunction is an endophenotype of ADHD symptoms [6], resulting in multiple complications in childhood, such as being overweight and obese [7] and motor impairments [8]. Considering the significance of EF development among children [4], childhood can be regarded as a highly critical period to improve EFs.

Although motor deficits are not a primary symptom of ADHD, many studies have indicated that relative to their typically developing (TD) peers, children with ADHD underperform in assessments of motor abilities, which include fine motor skills, gross motor skills, and static and dynamic balance [9]. Deficits in motor skills may affect PA [10], physical fitness [11], activities of daily living [12], and quality of life [10], and they may result in obesity [13], thereby affecting physical and psychological health and behavioral outcomes. Furthermore, Liang et al. [14] found the significant differences in motor proficiency between children with and without ADHD. Cho et al. [15] revealed no significant age-related differences on motor performance between children with and without ADHD, indicating that children with motor difficulties may continue to experience the same challenges into adolescence. Therefore, implementing appropriate motor-based interventions is recommended.

Physical activities have been recognized as a promising alternative or adjunctive treatment, as they are often associated with improvements in symptoms, EFs, and motor skills in individuals with ADHD [16]. Multiple studies have examined the efficacy of different exercise interventions for individuals with ADHD, revealing positive outcomes [17]. For instance, swimming [18], aquatic exercise [19], exergaming [20], and table tennis [21] are examples of exercise interventions. All these exercise interventions, conducted over 8–12 weeks and involving various activities, were systematically designed to improve ADHD symptoms, EFs, and/or motor skills in children. These interventions differ in structure, cognitive demands, and physical intensity, thereby offering varied benefits for symptom management and developmental outcomes. However, these have not yet been thoroughly vetted against established evidence based interventions.

Although various physical activities have demonstrated benefits for children with ADHD, inline skating remains underexplored in this population. Prior studies have mainly focused on TD children [22], leaving a gap in understanding its therapeutic potential. Inline skating uniquely combines aerobic exercise with cognitive and motor demands, engaging EFs such as working memory, cognitive flexibility, and inhibitory control [23]. It also targets motor domains often delayed in children with ADHD, including balance, coordination, and postural control [24]. The activity's rhythmic and dynamic nature may support emotional regulation and reduce hyperactivity by regulating dopamine and norepinephrine levels [25]. Unlike more conventional sports, inline skating has a novelty and thrill factor that appeals to children with ADHD, who often struggle with task persistence. Its novelty and sense of mastery can further enhance motivation. Together, these features make inline skating a promising, integrative approach for ADHD intervention.

Therefore, the aim of this study was to examine the impact of a 12-week inline skating intervention on EFs as the primary outcome, and on symptoms and motor proficiency as secondary outcomes, in two groups of children with ADHD aged 6–12 years. We hypothesize that (a) a 12-week inline skating intervention would lead to significant improvements in ADHD symptoms, EFs, and motor proficiency in both groups of children with ADHD and (b) the intervention effect would sustain for at least 12 weeks in the primary-grouping children with ADHD.

## 2. Methods

### 2.1. Design and Procedures

This study received ethical approval from the Research Ethics Committee of the National Changhua University of Education (Approval No. 103-085) according to the Declaration of Helsinki. The parents of children with ADHD provided written informed consent, and children provided assent to participate. Using a matched-pair randomization, all participants were screened for eligibility and matched on their age, medication status, and ADHD presentation before randomly assigned to one of the two groups: intervention group (IG) (baseline (Time 1), posttest (Time 2), and follow-up (Time 3)) and wait-list control group (CG) (control condition (from Time 1 to Time 2) and intervention condition (from Time 2 to Time 3)).

### 2.2. Participants

Participants were recruited through relevant nongovernmental organizations, information dissemination in various elementary schools, and word-of-mouth among parents. The inclusion criteria were as follows: (a) being a child aged between 6 and 12 years; (b) having ADHD diagnosed through the use of a diagnostic interview and questionnaires in accordance with the *Diagnostic and Statistical Manual of Mental Disorders, Fourth Edition (DSM-IV)*, as provided by a pediatrician or psychiatrist; and (c) having no additional disabilities or diseases (e.g., physical conditions and psychiatric comorbidity) that may interfere with the intervention, EF assessments, or motor proficiency evaluations.

In addition to the formal diagnosis made by a pediatrician or psychiatrist, the primary caregiver confirmed the presence of the severity of ADHD symptoms a priori by using the Chinese version of the ADHD test [26], and the Chinese version of the Child Behavior Checklist [27]. None had multiple co-occurring conditions. Parents were instructed to keep the usual treatment consistent, including the type and dosage of medication, throughout the intervention. As a result, we did not control the typical treatment methods or the medications and dosages provided to the children.

A power analysis was conducted using G⁣^∗^Power for a 2 × 2 mixed-design analysis of variance (ANOVA) (group × time), with an estimated effect size of *f* = 0.61 (converted from *η_p_*^2^ = 0.26), *α* = 0.05, and power = 0.80. The analysis indicated that a total sample size of *n* = 49 would be required to achieve adequate power. However, due to feasibility constraints, we recruited *n* = 24 participants (12 per group), which may limit the ability to detect smaller effects. As shown in Table 1, no differences were observed between groups in demographic data at baseline (all *p* > 0.05).

### 2.3. Intervention

The intervention comprised a 12-week inline skating exercise focusing on a set of inline skating-based activities targeting an array of behavioral and neurocognitive domains including inhibitory control (e.g., “Simon Says,” freeze dance), working memory (e.g., finding “hidden treasures” under cones, recalling previously learned skills), motor control (e.g., variations of inline skating skills), and cardiovascular endurance training, with a total of 24 sessions (two sessions per week). Each training session lasted 80 min, including 15 min of warm-up (jogging around the court, static stretching, and a race to equip skates and protective gear), 60 min of main activity training (divided into 40 min of motor skill training and 20 min of cardiovascular training), and 5 min of cooldown (including stretching and relaxation exercises, a session review with feedback, and a group cheer at the end of the class). During the second phase of intervention, the 40-min motor skill training covered 20 essential basic inline skating skills: (a) marching in a standing position, (b) properly falling and getting up, (c) two-foot forward gliding, (d) marching while moving, (e) forward swizzling, (f) backward acceleration drills, (g) forward parallel turning, (h) forward snowplow stopping, (i) half swizzle pumps in a circle, (j) left and right forward balance turning, (k) forward dipping, (l) forward speed swizzling, (m) one-foot forward pushing, (n) two-foot backward gliding, (o) backward slaloming, (p) forward and backward half swizzle pumps in a circle, (q) backward swizzling, (r) forward full-squat gliding, (s) two-foot forward hopping, and (t) forward crossovers. The cardiovascular endurance training involved continuously skating around the perimeter of a large circle for 20 min, either clockwise or counterclockwise.

To ensure adherence to the intervention protocol, several strategies were implemented. Attendance was recorded at each session, and participants who missed sessions were contacted to encourage continued participation. Additionally, the principal researcher, instructors, and research assistants closely supervised all training sessions, ensuring that participants followed the prescribed inline skating exercises. The instructors provided verbal feedback and skill demonstrations to reinforce engagement. To assess session compliance, participants were asked to complete all designated exercises under supervision, and adjustments were made if necessary to maintain participation.

Before each weekly inline skating session, preclass discussions and postclass reviews were held by the principal researcher, main instructor, and research assistants to ensure a diverse program design that could improve the symptoms, EFs, and motor proficiency of children with ADHD. This inline skating intervention was conducted on the multipurpose ball court of the university gymnasium where the principal researcher worked. The main instructor had over a decade of experience in inline skating instruction and has also served as an expert coach for both the nation floorball and speed skating teams. In addition to the coach and principal researcher, six research assistants were recruited to assist with the inline skating program (all research assistants had experience in interacting with children with ADHD and were either undergraduate students of the Department of Physical Education or graduate students specializing in adapted physical education). The ratio of research assistants to study participants was 1:2.

### 2.4. Measures

Due to the confidentiality provisions of Taiwan's Special Education Law, the researchers were not given access to the children's individual intelligence quotient (IQ) scores. However, according to the school counselor, the IQ of children with ADHD fell within the range of 90–120, and they were all free of any serious developmental or psychiatric disorders. The following assessments were conducted by research staff who were trained by the principal researcher and were blind to participant treatment randomization.

#### 2.4.1. Chinese Version of the ADHD Test

The Chinese version of the ADHD test for ages 4–18 years and 11 months was used to assess severity of ADHD symptoms [26]. Three subtests are compiled based on the diagnostic criteria of DSM-IV: hyperactivity, impulsivity, and inattention. The questionnaire, with well-established reliability (range from 0.80 to 0.92), validity (content validity and criterion-related validity), and internal consistency (range from 0.88 to 0.95), consists of 36 items and was fill out by the parents of children [26].

#### 2.4.2. Stroop Color and Word Test (SCWT)

The children's version of the SCWT for ages 5–14 years was used to measure the children's ability to inhibit a response [28]. This version consists of three subtasks, namely, a “word” subtask, a “color” subtask, and a “color–word” subtask. Each subtask comprises 100 stimuli distributed evenly in a 5 (column) × 20 (row) matrix on a white 8.5 × 11-inch sheet of paper. In the first subtask, the words “red,” “green,” and “blue” are displayed in black 100 times in random order. In the second subtask, 100 solid-color rectangles printed in red, green, and blue are displayed. In the third subtask, 100 color–words are displayed in an incongruous color (e.g., the word “green” printed in red). The sequence of stimuli for these three subtasks is read from top to bottom (20 stimuli) and from left to right (5 columns), with participants aiming to complete as many as possible in the fastest and most accurate manner within a 45-s time limit (each subtask is performed once). Reported test–retest reliability coefficients for the children's Stroop test typically range from 0.71 to 0.89 [28].

Because the “word” and “color” subtests are not designed to measure inhibitory control, only the raw scores of the “color–word” subtask were used in statistical analysis in this study for examining the inhibitory response capabilities of children with ADHD.

#### 2.4.3. Spatial Working Memory (SWM)

An SWM test obtained from the Cambridge Neuropsychological Test Automated Battery (CANTAB) was used. The CANTAB is more reliable and valid than many traditional clinical assessments [29]. The validity of the CANTAB has been supported by numerous studies across various psychiatric populations, including individuals with ADHD [30]. This test begins with a number of colored boxes shown on a screen. An increasing number of boxes are subsequently presented on the screen during each trial. During the test, participants are instructed to search for hidden tokens, to open the boxes by touching them, and not to return to a box that has already yielded a token (tokens are not allowed to appear in a previous location within a single trial).

The analyzed measures are as follows: (a) total errors (number of total errors (i.e., number of times a subject touches a box that is certain not to contain a token) calculated using the between errors (between searches, i.e., whether they revisited a box where a blue token had already been found), within errors (within searches, i.e., whether they revisited a box known to be empty), and double errors (as some touch responses can be both errors made within and between searches) of particular box problems, that is, between errors + within errors − double errors) and (b) strategy (number of times the participant started a new search by touching a different box).

#### 2.4.4. Bruininks–Oseretsky Test of Motor Proficiency-2 (BOT-2)

The BOT-2 is appropriate for aged 4–21 years to evaluate the fine and gross motor proficiency [31]. This assessment comprises a total of 53 test items that are organized into four composite scales. Each scale is further divided into two subtests, each comprising five to nine test items: (a) fine manual control (FMC), which comprises fine motor precision and fine motor integration; (b) manual coordination (MC), which comprises manual dexterity and upper limb coordination; (c) body coordination (BC), which comprises balance and bilateral coordination; and (d) strength and agility (SA), which comprises strength and running speed and agility. The raw scores of these eight subtests are converted into point scores, which are subsequently summed to yield a total point score. This total point score can subsequently be transformed into a scale score. These scale scores can be compared with normative data to further convert them into standard scores for the four composite scales: FMC, MC, BC, and SA. The sum of the standard scores for these four composites provides the total motor composite (TMC) score. Both the validity and reliability of the BOT-2 have been established for individuals with developmental coordination disorders, mild-to-moderate intellectual disabilities, high-functioning autism, and Asperger's syndrome. Regarding subtest internal consistency reliability, BOT-2 exhibits average reliability ranging from 0.70 to 0.80 across three age groups: 4–7 years, 8–11 years, and 12–21 years. The internal composite consistency reliability of the BOT-2 ranges from 0.80 to 0.90, and its test–retest reliability varies from 0.69 to 0.80. Its interrater reliability also ranges between 0.92 and 0.99 [31].

### 2.5. Statistical Analysis

All statistical analyses were conducted using IBM SPSS Statistics Version 25 (IBM, Armonk, New York, United States). Participant characteristics, namely, age, height, weight, and body mass index (BMI) were compared using an independent *t* test. To evaluate the effects of the intervention, ANOVA with a 2 (Time 1 vs. Time 2) × 2 (group: IG vs. wait-list CG) mixed-model design was used to measure symptoms, EFs, and motor proficiency outcomes. The Bonferroni correction was used to adjust *p* values for multiple comparisons in post hoc analyses. Simple main effects were determined if any interaction effect was deemed significant. Effect size was reported as a partial eta-squared (*η*_*p*_^2^) value in ANOVA, with effects interpreted as small (0.01), medium (0.06), or large (0.14). After conducting the ANOVA, paired-sample *t*-tests were performed to evaluate the effects of the inline skating program in wait-list CG (Time 2 vs. Time 3) and the maintenance effects in IG (Time 2 vs. Time 3). The effect size was reported as Cohen's *d* for the paired *t*-tests. A *p* value less than 0.05 was considered statistically significant.

## 3. Results

All participants completed every assessment at each time point, and no missing data were recorded. The participation rates were comparable between the IG (93.25% ± 4.52%) during the first 12-week phase and the CG (94.00% ± 4.33%) in the second 12-week phase (*t* = −0.42, *p* = 0.679). Additionally, every participant fully completed the 12-week inline skating exercise program. At baseline (Time 1), there were no significant differences in outcome variables between the groups (all *p* > 0.05) (Table 2).

### 3.1. Effects of the Intervention on ADHD Symptoms

**Figure 1:**
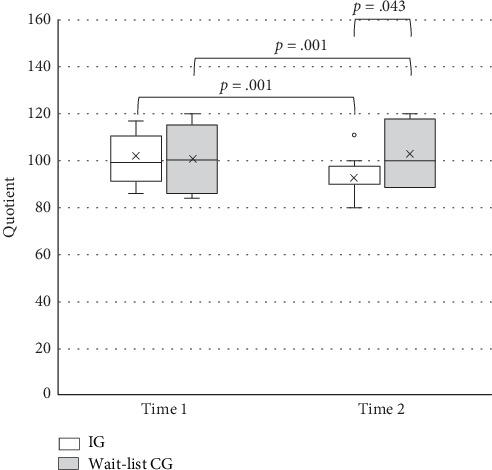
ADHD quotient of two groups of children with ADHD before (Time 1) and after (Time 2) intervention.

Table 3 lists the results of two-way ANOVA with a mixed factorial design. ANOVA revealed a significant main effect of time on ADHD quotient. No significant main effect of group was observed. A significant time-by-group interaction effect was revealed. After the intervention, improvements in ADHD symptoms were observed in the IG in comparison with the wait-list CG (*F* = 4.59, *p* = 0.043; Figure 1). The IG showed improvements in ADHD symptoms over time (*F* = 22.71, *p* = 0.001). The wait-list CG showed a poor performance in the quotient score at Time 2 compared to Time 1 (*F* = 18.56, *p* = 0.001).

Changes in ADHD quotient for the IG remained stable, with differences between Time 2 and Time 3 (follow-up assessment) being nonsignificant. However, the ADHD quotient at Time 3 differed significantly in the wait-list CG compared with that of at Time 2 (intervention condition; *t* = 7.06, *p* < 0.001, Cohen's *d* = 0.56), suggesting a significant improvement after the intervention.

### 3.2. Effects of the Intervention on EFs

Significant main effects of time on all EFs were identified (Table 3). All participants exhibited significantly better performance at Time 2 than at Time 1. A significant main effect of group was only noted for the color–word score. Significant time-by-group interaction effects were observed for all EFs.

**Figure 2:**
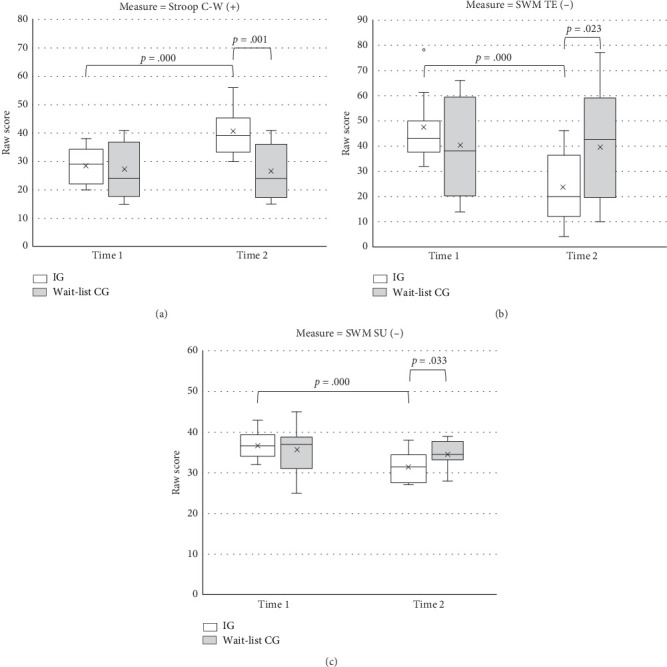
(a) The Stroop C-W outcomes for two groups of children with ADHD before (Time 1) and after the intervention (Time 2); C-W = color and word. (b) The SWM TE for two groups of children with ADHD before (Time 1) and after the intervention (Time 2); SWM = spatial working memory; TE = total errors. (c) The SWM SU outcomes for two groups of children with ADHD before (Time 1) and after the intervention (Time 2); SWM = spatial working memory; SU = strategy utilization.

Follow-up of the simple main effect revealed improvements on the EFs of the IG (higher color–word scores, *F* = 125.17, *p* < 0.001; fewer total errors, *F* = 65.53, *p* < 0.001; more favorable strategy utilization, *F* = 75.94, *p* < 0.001) after the intervention (Figure 2). The performance was better in the IG than in the wait-list CG after the intervention (color–word, *F* = 15.98, *p* = 0.001; total errors, *F* = 5.93, *p* = 0.023; strategy utilization, *F* = 5.17, *p* = 0.033).

Comparison between Time 2 and Time 3, the IG did not reveal a significant difference in any of the outcomes of EFs, whereas the wait-list CG performed significantly better on the color–word (*t* = −4.00, *p* = 0.002, Cohen's *d* = −0.76), total errors (*t* = 4.89, *p* < 0.001, Cohen's *d* = 0.80), and strategy utilization (*t* = 2.59, *p* = 0.025, Cohen's *d* = 0.88) at Time 3 than at Time 2, indicating a greater improvement after the intervention.

### 3.3. Effects of the Intervention on Motor Proficiency

ANOVA revealed a significant main effect of time on all composites except FMC (Table 3). The scores obtained at Time 2 were significantly higher than those obtained at Time 1. A significant main effect of group was only observed for MC composite. Significant time-by-group interaction effects were observed for all outcome variables.

**Figure 3:**
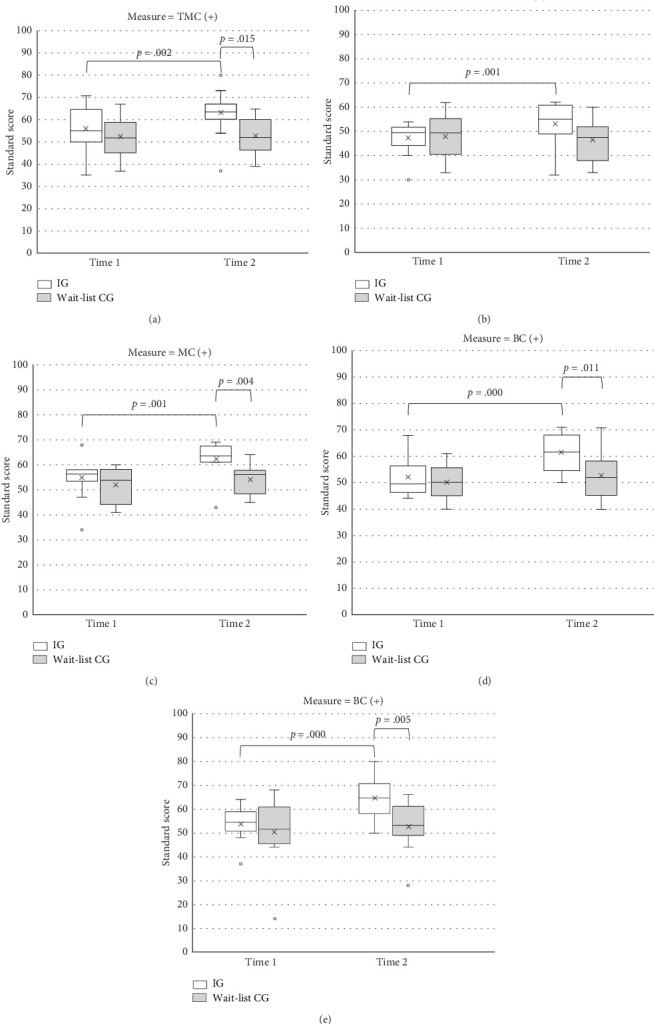
(a) Motor proficiency (TMC) outcomes for two groups of children with ADHD before (Time 1) and after the intervention (Time 2); TMC = total motor composite. (b) Motor proficiency (FMC) outcomes for two groups of children with ADHD before (Time 1) and after the intervention (Time 2); FMC = fine manual control. (c) Motor proficiency (MC) outcomes for two groups of children with ADHD before (Time 1) and after the intervention (Time 2); MC = manual coordination. (d) Motor proficiency (BC) outcomes for two groups of children with ADHD before (Time 1) and after the intervention (Time 2); BC = body coordination. (e) Motor proficiency (SA) outcomes for two groups of children with ADHD before (Time 1) and after the intervention (Time 2); SA = strength and agility.

Follow-up of the simple main effects revealed that the IG had a significantly better performance on TMC (*F* = 7.02, *p* = 0.015), MC (*F* = 10.69, *p* = 0.004), BC (*F* = 7.68, *p* = 0.011), and SA (*F* = 9.95, *p* = 0.005) after the intervention compared with the wait-list CG (Figure 3). IG had a significantly higher score after the intervention compared with Time 1 (TMC, *F* = 17.40, *p* = 0.002; FMC, *F* = 19.56, *p* = 0.001; MC, *F* = 22.30, *p* = 0.001; BC, *F* = 40.29, *p* < 0.001; SA, *F* = 128.66, *p* < 0.001). By contrast, the wait-list CG exhibited similar performance on all BOT-2 outcomes at Time 2 compared with Time 1.

For wait-list CG, the comparison between Time 2 and Time 3 revealed significant differences in TMC (*t* = −2.53, *p* = 0.002, Cohen's *d* = −0.51), MC (*t* = −2.27, *p* = 0.045, Cohen's *d* = −0.67), BC (*t* = −3.09, *p* = 0.010, Cohen's *d* = −0.76), and SA (*t* = −3.00, *p* = 0.012, Cohen's *d* = −0.58), indicating a significant improvement in scores after intervention. For IG, a comparison between Time 2 and Time 3 revealed no significant difference in BOT-2 outcomes, suggesting that intervention effects were sustained.

## 4. Discussion

In this study, we examined the effects of an inline skating intervention on the symptoms, EFs, and motor proficiency of children with ADHD. The results indicated that the intervention improved the children's symptoms, EFs, and motor proficiency and that improvements in children with ADHD could persist in the long term.

Regarding the motor proficiency training, our findings are consistent with previously conducted RCTs of similar interventions indicating that motor-based interventions in general seem to improve motor skills in children with ADHD [16]. This inline skating training program is based on the rationale that motor skills mastery has a positive link to executive functioning, like others [21, 32]. Such interventions use a task-oriented approach and are, therefore, meaningful for the child. Inline skating requires balance, muscle tension, BC, and various complex movement skills. In the present study, the first stage of the intervention involved 15 min of warm-up, including a specially designed competition for putting on shoes and protective gear, enabling children to practice donning accessories every time, and hence, improving their hand coordination (FMC and MC). The second stage of the intervention involved 60 min of main activity training, divided into 40 min of basic practice of movement skills with inline skating and 20 min of movement skill practice as a part of cardiovascular endurance training. This structure enabled children with ADHD to consistently practice movement skills of inline skating at their own pace. By learning these inline skating movement skills, children with ADHD not only enhanced their BC but also developed movement skill elements pertaining to SA. To achieve these goals, children with ADHD should master inline skating techniques such as two-foot forward gliding, forward swizzling, forward speed swizzling, forward dipping, forward full-squat gliding, two-foot forward hopping, and forward crossovers. Performing these techniques involves complex actions such as pushing feet together side by side, squatting, propulsive pushing, and jumping. This type of training not only improves basic movement skills of inline skating among children but also promotes comprehensive development in MC, BC, SA, and overall motor proficiency (i.e., TMC). The third stage of the intervention involved 5 min of cooldown, including a verbal review of the day's lesson and a reemphasis on the key movement skills learned on that day, thus enabling children with ADHD to review their learned movement skills on their own. Overall, these aspects of training methodologies may explain why the children with ADHD experienced improvements in FMC, MC, BC, SA, and overall TMC after the intervention.

The EF results of the current study are consistent with the previous narratives of Liang et al. [33] and Huang et al. [17]b, indicating that prolonged exercise interventions have the beneficial impacts on improving EFs in the ADHD population. Furthermore, Qiu et al. [17]a indicated that what exercise intervention significantly improved EFs in ADHD population may be due to the inclusion of studies with moderate to above moderate exercise intensity. In the inline skating exercise implemented in this study, the second part of the intervention comprised 60 min of main activity training, which specifically included 20 min of continuous clockwise or counterclockwise skating around the perimeter of the gymnasium. The participants practiced basic inline skating movement skills at their own pace while simultaneously engaging in progressive cardiovascular endurance training. Cardiorespiratory fitness is positively correlated with neuroelectric indices of attention, working memory, and reaction speed [34]. The present inline skating intervention had significant impact on EFs may be because participants with ADHD were in a stable and predictable environment, allowing them to pace themselves and experience a lower cognitive load. As a result, participants with ADHD were better able to acquire motor skills and maintain moderate intensity, resulting in increased cardiorespiratory fitness. Future research must be conducted with greater rigor and on a larger scale to substantiate this argument.

The current inline skating exercise intervention significantly improved EF subdomains, including inhibitory control and working memory. This finding is consistent with the results of intervention type such as swimming, running, and weightlifting, but inconsistent with those of intervention type such as table tennis, judo, and taekwondo [17]a. In our design, we focused on the transition of various movement skills across spaces to enable children with ADHD to fully utilize their SWM capacity while practicing movement skills. For example, we constructed a training route with a zigzag pattern, requiring the children to shift to another inline skating skill at each transition point, followed by the practice of new movement skills (i.e., SWM training). Before each practice session, the instructors provided verbal cues and demonstrated the correct movements to ensure that participants remembered the movement skills that they were supposed to execute and to ensure that they followed the planned route (i.e., space). Although variations in the type, duration, and frequency of exercise may explain certain differences between studies, future research should broaden the ADHD population for which exercise interventions are appropriate as well as subdividing the beneficial effects of exercise interventions on EF subfunctions.

The ADHD symptoms ameliorated in the current study. This seems in line with the limited studies on the effects of physical exercise interventions on core ADHD symptoms [35]. In contrast to most studies that were included in the meta-analyses [36], evidence for the effects of physical exercise interventions was highly suggestive for inhibitory control but weak for working memory outcome in children with ADHD. Skogli et al. [37] detected that improvements in ADHD symptoms over time were only linked to improvements in working memory. While the current study found improvements in working memory performance, it remains unclear whether this change reflects a direct impact on ADHD symptoms.

The value of outdoor pursuits such as inline skating in physical education and PA has been well documented [38]. Inline skating is one of the sports that the “exercise for all” policy of Taiwanese government vigorously promotes. It can freely adjust the intensity and time of exercise according to individual needs or learning levels. Compared with swimming, table tennis, basketball and other exercises, the sports venue space of inline skating is less restricted. As such, inline skating exercise seems to represent a feasible way of improving EFs, symptoms, and motor proficiency in children with ADHD who may not feel comfortable in an organized or competitive atmosphere.

## 5. Conclusion

Inline skating intervention seems to have long-term beneficial effects in children with ADHD. While the observed improvements in ADHD symptoms, EFs, and motor proficiency were statistically significant, the magnitude of change was moderate to small in some measures. Future research should examine long-term follow-ups, larger samples, and additional measures of functional impact to better assess the practical significance of these changes.

### 5.1. Limitations

This study has some limitations. First, our participants comprised only elementary school children, with limitations in terms of sample size (*n* = 24), age range (6–12 years), and gender (male). Second, we did not control for medication use and whether medication status changed for any of the participant before the endpoint. Third, our assessment of motor proficiency and EFs was limited to only three tests: BOT-2, SCWT, and SWM. Fourth, the exercise intensity of inline skating exercise was not measured. Fifth, a more appropriate CG (e.g., a different sport) should be considered. Sixth, ADHD symptom severity was assessed solely through parent reports, which may be prone to rater bias. Finally, the actual effect sizes are not large, which suggests limited clinical relevance.

## Figures and Tables

**Table 1:** Participant descriptive characteristics.

	**Intervention group (** **n** = 12**)**	**Wait-list control group (** **n** = 12**)**	**t**	**p**
	**M** ± **SD**	**M** ± **SD**		
Age (years)	10.21 ± 1.27	10.21 ± 1.24	0.02	0.999
Height (cm)	137.96 ± 5.56	138.02 ± 8.68	−0.20	0.985
Weight (kg)	32.83 ± 7.98	36.84 ± 12.04	−0.96	0.346
BMI (kg/m^2^)	17.13 ± 3.30	18.94 ± 4.14	−1.19	0.247
Medication (*n*, %)	2 (17%)	2 (17%)		
ADHD presentation (*n*, %)				
Hyperactivity	3 (25%)	3 (25%)		
Inattentiveness	4 (33%)	4 (33%)		
Combined	5 (42%)	5 (42%)		
ADHD Q (*n*, %)				
≥ 131	0 (0%)	0 (0%)		
121–130	0 (0%)	0 (0%)		
111–120	3 (25%)	3 (25%)		
90–110	7 (58%)	5 (42%)		
80–89	2 (17%)	4 (33%)		
70–79	0 (0%)	0 (0%)		
≤ 69	0 (0%)	0 (0%)		

*Note:* The higher the quotient lead levels, the more severe.

Abbreviations: ADHD = attention deficit hyperactivity disorder, BMI = body mass index, Q = quotient.

**Table 2:** ADHD symptoms, executive function, and motor proficiency by the group at three assessments.

	**Time 1**		**Intervention group (** **n** = 12**)**			**Wait-list control group (** **n** = 12**)**		
	**t**	**p**	**Time 1**	**Time 2**	**Time 3**	**Time 1**	**Time 2**	**Time 3**
ADHD Q (−)	0.21	0.833	100.67 ± 10.86	92.67 ± 9.98	94.08 ± 11.01	99.58 ± 13.82	102.58 ± 12.54	95.75 ± 11.92
Stroop								
C-W (+)	0.64	0.532	28.50 ± 6.25	40.33 ± 8.28	37.67 ± 8.54	26.42 ± 9.48	26.17 ± 9.06	33.50 ± 10.16
SWM								
TE (−)	1.14	0.271	46.67 ± 12.56	23.33 ± 13.58	21.92 ± 17.94	39.08 ± 19.44	41.50 ± 21.99	25.17 ± 18.63
SU (−)	0.47	0.644	36.50 ± 3.23	31.42 ± 3.82	32.08 ± 3.99	35.58 ± 5.93	34.67 ± 3.14	30.00 ± 6.82
BOT-2								
TMC (+)	0.97	0.342	55.67 ± 9.97	62.83 ± 10.47	61.50 ± 10.91	51.83 ± 9.34	52.58 ± 8.37	56.75 ± 7.82
FMC (+)	−0.28	0.781	46.92 ± 6.76	52.75 ± 9.31	49.67 ± 8.92	47.83 ± 9.04	46.58 ± 8.98	46.75 ± 9.62
MC (+)	0.88	0.391	54.75 ± 8.06	62.67 ± 6.85	61.58 ± 7.63	52.08 ± 6.82	54.08 ± 5.98	59.92 ± 10.85
BC (+)	0.71	0.486	51.83 ± 7.28	61.42 ± 7.23	59.00 ± 8.55	49.83 ± 6.51	52.33 ± 8.75	59.25 ± 9.39
SA (+)	0.77	0.451	53.92 ± 7.04	64.50 ± 8.04	63.50 ± 8.40	50.50 ± 13.72	52.58 ± 10.33	57.75 ± 7.19

*Note:* (+) = higher scores represent better performance; (−) = lower scores represent better performance.

Abbreviations: ADHD = attention deficit hyperactivity disorder; BC = body coordination; *BOT-2* = *Bruininks–Oseretsky Test of Motor Proficiency, Second Edition*; C-W = color-word; FMC = fine manual control; MC = manual coordination; Q = quotient; SA = strength and agility; SU = strategy utilization; SWM = spatial working memory; TE = total errors; TMC = total motor composite.

**Table 3:** Summary of the two-way (time × group) ANOVA with repeated measures on one factor (time).

	**Time (T)**		**Group (G)**		**T** × **G**		**Simple main effect tests**	
	**F** ** (1, 22)**	**η**_**p**_^2^	**F** ** (1, 22)**	**η**_**p**_^2^	**F** ** (1, 22)**	**η**_**p**_^2^	**Group difference at T2**	**Time difference by group**
ADHD Q (−)	7.59⁣^∗^	0.26	0.86	0.04	36.63⁣^∗∗^	0.63	IG < wait-list CG	IG: T1 > T2; Wait-list CG: T1 < T2
Stroop								
C-W (+)	62.38⁣^∗∗^	0.74	5.94⁣^∗^	0.213	67.88⁣^∗∗^	0.76	IG > wait-list CG	IG: T1 < T2
SWM								
TE (−)	24.64⁣^∗∗^	0.53	0.61	0.03	37.35⁣^∗∗^	0.63	IG < wait-list CG	IG: T1 > T2
SU (−)	9.11⁣^∗∗^	0.29	0.70	0.03	4.39⁣^∗^	0.17	IG < wait-list CG	IG: T1 > T2
BOT-2								
TMC (+)	16.48⁣^∗∗^	0.43	3.47	0.14	10.83⁣^∗∗^	0.33	IG > wait-list CG	IG: T1 < T2
FMC (+)	3.67	0.14	0.58	0.03	8.76⁣^∗∗^	0.29	—	IG: T1 < T2
MC (+)	22.90⁣^∗∗^	0.51	4.51⁣^∗^	0.17	8.15⁣^∗∗^	0.27	IG > wait-list CG	IG: T1 < T2
BC (+)	39.83⁣^∗∗^	0.64	3.64	0.14	13.69⁣^∗∗^	0.38	IG > wait-list CG	IG: T1 < T2
SA (+)	41.38⁣^∗∗^	0.65	3.66	0.14	18.63⁣^∗∗^	0.46	IG > wait-list CG	IG: T1 < T2

*Note:η*_*p*_^2^ = partial eta-squared.

Abbreviations: ADHD = attention deficit hyperactivity disorder; BC = body coordination; *BOT-2* = *Bruininks–Oseretsky Test of Motor Proficiency, Second Edition*; CG = control group; C-W = color-word; FMC = fine manual control; IG = intervention group; MC = manual coordination; Q = quotient; SA = strength and agility; SU = strategy utilization; SWM = spatial working memory; TE = total errors; TMC = total motor composite.

⁣^∗^*p* < 0.05; ⁣^∗∗^*p* < 0.01.

## Data Availability

The datasets analyzed in this study are not publicly available due to data protection requirements. However, anonymized data or files may be available from the corresponding authors upon reasonable request.
